# Health workers’ perception vs medically approved COVID-19 infection risk: the risk communication gap

**DOI:** 10.1093/eurpub/ckac129.176

**Published:** 2022-10-25

**Authors:** E Kuhlmann, GMN Behrens, A Cossmann, S Homann, C Happle, A Dopfer-Jablonka

**Affiliations:** Clinic for Rheumatology and Immunology, Medizinische Hochschule Hannover, Hannover, Germany

## Abstract

**Background:**

This study analyses how healthcare workers (HCWs) perceived risks, protection and preventive measures during the COVID-19 pandemic in relation to medically approved risks and organisational measures. We aim to explore ‘blind spots’ of pandemic protection and identify mental health needs.

**Methods:**

A German multi-method hospital study at Hannover Medical School serves as an ‘optimal-case’ scenario of a high-income country, well-resourced hospital sector and an organisation with low HCW infection rate serves to explore governance gaps in HCW protection. Document analysis, expert information and survey data (n = 1163) were collected as part of a clinical study into SARS-CoV-2 serology testing during the second wave of the pandemic (November 2020-February 2021). Selected survey items included perceptions of risks, protection and preventive measures. Descriptive statistical analysis and regression were undertaken for gender, profession and COVID-19 patient care.

**Results:**

Our study reveals a low risk of 1% medically approved infections among participants, but a much higher mean personal risk estimate of 15%. The majority (68.4%) expressed ‘some’ to ‘very strong’ fear of acquiring infection at the workplace. Individual protective behaviour and compliance with protective workplace measures were estimated as very high. Yet only about half of the respondents felt strongly protected by the employer; 12% even perceived ‘no’ or ‘little’ protection. Gender and contact with COVID-19 patients had no significant effect on the estimations of infection risks and protective workplace behaviour, but nursing was correlated with higher levels of personal risk estimations and fear of infection.

**Conclusions:**

A strong mismatch between low medically approved risk and personal risk perceptions of HCWs brings stressors and threats into view, that may be preventable through improved information, risk communication and inclusion of mental health support in pandemic preparedness.

**Key messages:**

• Healthcare workers’ perceptions of COVID-19 infection risks are much higher than medically approved infection risk.

• Pandemic preparedness and protection plans must pay greater attention to information, risk communication and mental health needs.

